# Successful termination of ventricular tachycardia by intrinsic antitachycardia pacing delivered from a left bundle branch area pacing lead: a case report

**DOI:** 10.1093/ehjcr/ytag577

**Published:** 2026-07-30

**Authors:** Noriaki Moriyama, Yoichi Sugiyama, Yohei Hiruma, Kunihiko Shimizu, Shigeru Saito

**Affiliations:** Department of Cardiology, Shonan Kamakura General Hospital, 1370-1 Kamakura Okamoto, Kamakura 2478533, Japan; Department of Cardiology, Shonan Kamakura General Hospital, 1370-1 Kamakura Okamoto, Kamakura 2478533, Japan; Department of Cardiology, Shonan Kamakura General Hospital, 1370-1 Kamakura Okamoto, Kamakura 2478533, Japan; Department of Cardiology, Shonan Kamakura General Hospital, 1370-1 Kamakura Okamoto, Kamakura 2478533, Japan; Department of Cardiology, Shonan Kamakura General Hospital, 1370-1 Kamakura Okamoto, Kamakura 2478533, Japan

**Keywords:** Ventricular tachycardia, Intrinsic antitachycardia pacing (iATP), Left bundle branch area pacing (LBBAP), Left bundle branch optimized–CRT (LOT-CRT), Case report

## Abstract

**Background:**

Intrinsic antitachycardia pacing (iATP) is an advanced algorithm designed to optimize pacing sequences for ventricular tachycardia (VT) termination. Although left bundle branch area pacing (LBBAP) has emerged as a physiological pacing strategy and is increasingly incorporated into cardiac resynchronization therapy, its role as a delivery site for iATP remains unclear.

**Case summary:**

We report a 57-year-old man with advanced heart failure who underwent implantation of LBB–optimized cardiac resynchronization therapy with defibrillator for secondary prevention. During follow-up, multiple episodes of sustained VT with different morphologies were consistently and successfully terminated by iATP delivered exclusively from the LBBAP lead, without the need for conventional ATP or shock therapies.

**Discussion:**

This case suggests that conduction system pacing via the LBB area may provide a novel and effective substrate for antitachycardia pacing, potentially enabling more rapid engagement of re-entrant circuits through the His–Purkinje system. Further studies are warranted to establish the efficacy of iATP delivered from conduction system pacing leads.

Learning points

- Intrinsic antitachycardia pacing (iATP) from the left bundle branch area is feasible and represents a physiologically favourable alternative for ventricular tachycardia termination.
- Compared with RV-ATP, left bundle branch (LBB) area-based ATP may enable faster His–Purkinje engagement and improve efficacy, especially in septal or left ventricular circuits.
- With increasing LBB–optimized cardiac resynchronization therapy with defibrillator use, optimal ATP programming, sensing, and long-term safety of LBB area-based ATP require further study.

## Introduction

Intrinsic antitachycardia pacing (iATP) is an automated ATP algorithm designed to optimize pacing sequences for the termination of ventricular tachycardia (VT) by dynamically adjusting pacing intervals based on the response to prior stimuli. Compared with conventional ATP, iATP may improve VT termination while reducing unnecessary shock therapy.^1^ Recently, left bundle branch area pacing (LBBAP) has emerged as a physiological pacing strategy and has been increasingly incorporated into cardiac resynchronization therapy systems.^2^ In particular, LBB–optimized cardiac resynchronization therapy with defibrillator (LOT-CRTD) has been proposed as a novel approach to achieve physiological resynchronization while maintaining defibrillation capability.^3^ However, the effectiveness of iATP delivered from an LBBAP lead has not been reported, and its role in patients treated with LOT-CRTD remains unknown. We report a case in which sustained VT was successfully terminated by iATP delivered from an LBBAP lead in a patient with LOT-CRTD.

## Summary figure

**Figure:**
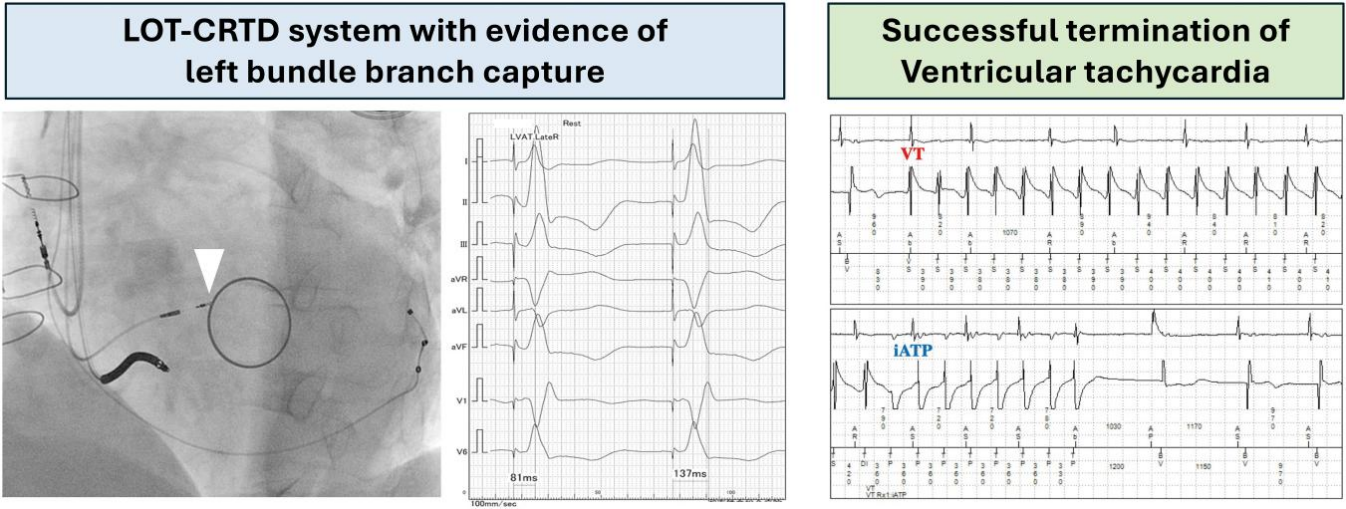
Successful intrinsic antitachycardia pacing from left bundle branch area pacing lead. White arrowhead: left bundle branch area pacing lead. LOT-CRTD, left bundle branch area pacing–optimized cardiac resynchronization therapy with defibrillator.

## Case presentation

**Figure 1:**
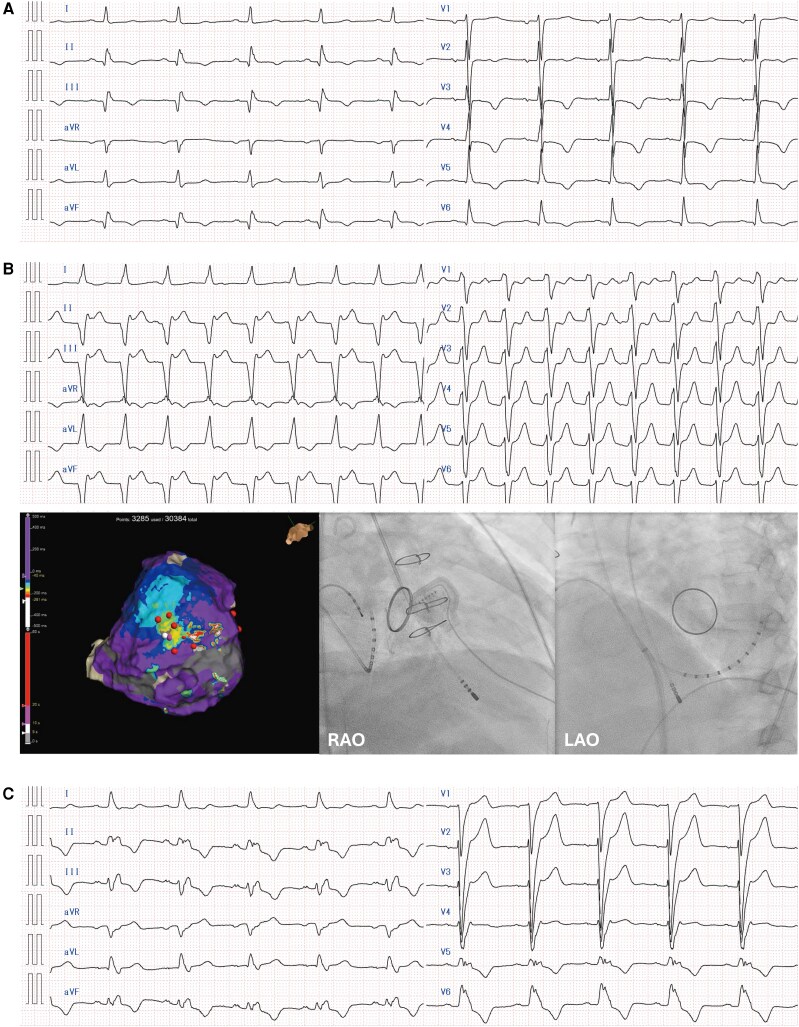
(*A*) 12-lead electrocardiogram after removal of Impella CP. Sinus rhythm, QRS duration 106 ms with abnormal Q wave in II, III, aVF. (*B*) 12-lead electrocardiogram demonstrating VT, HR 107 beats/min, QRS duration 156 ms. Electroanatomical mapping identified the VT origin at the septal aspect of the left ventricular apex, and radiofrequency ablation was successfully performed. (*C*) Iatrogenic left bundle branch block pattern on the 12-lead electrocardiogram. Sinus rhythm, QRS duration 196 ms.

A 57-year-old man (height 175 cm, weight 92 kg, body surface area 2.07 m^2^) with a history of advanced heart failure with reduced ejection fraction was referred to our institution. His medical history included coronary artery bypass grafting, mitral valve replacement, tricuspid annuloplasty, and surgical closure of an atrial septal defect. He also had a prior episode of ventricular fibrillation requiring resuscitation following cardiac surgery. At the time of presentation, the patient’s medications included furosemide 40 mg/day, enalapril maleate 2.5 mg/day, spironolactone 25 mg/day, amiodarone 100 mg/day, warfarin 3 mg/day, rosuvastatin 5 mg/day, and aspirin 100 mg/day. The patient presented with syncope and cardiogenic shock due to sustained VT. Mechanical circulatory support with an Impella CP (Abiomed, Danvers, MA, USA) was initiated because of low-output syndrome (left ventricular ejection fraction 15%) even after VT termination by defibrillation. After haemodynamic stabilization and subsequent removal of the Impella device, slow VT recurred (heart rate (HR) 107 beats/min, QRS 156 ms), leading to worsening heart failure. Therefore, catheter ablation was performed. Electroanatomical mapping identified the VT origin at the septal aspect of the left ventricular apex, and radiofrequency ablation was successfully performed at this site. Following the ablation procedure, the patient developed a new and persisted left bundle branch (LBB) block pattern on the electrocardiogram (PR 178 ms, QRS 196 ms) (*Figure 1*). Given the history of ventricular fibrillation and sustained VT, implantation of a defibrillator for secondary prevention was indicated. Although conventional cardiac resynchronization therapy with defibrillator (CRT-D) was considered, LOT-CRTD was selected to achieve more physiological ventricular activation through direct engagement of the conduction system and to maximize the potential for electrical and mechanical resynchronization in this relatively young patient with advanced heart failure.

**Figure 2:**
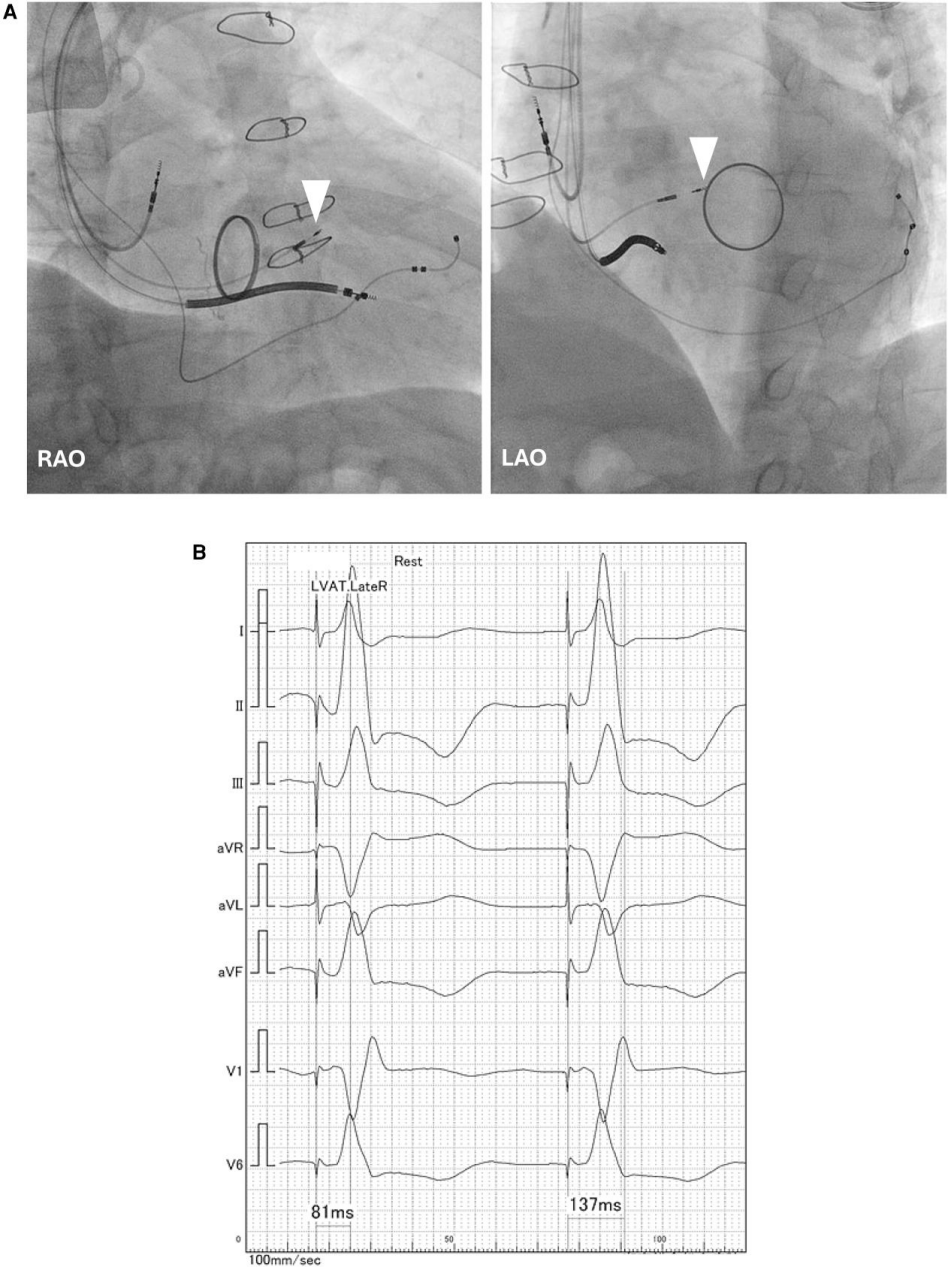

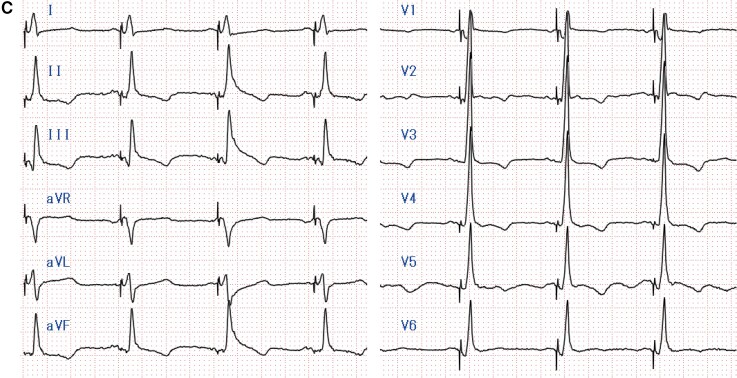
(*A*) LOT-CRTD system (white arrowhead: LBBAP lead). (*B*) Intra-procedural electrocardiogram showing the evidence of LBB capture. (*C*) 12-lead electrocardiogram demonstrating biventricular pacing with paced QRS duration of 124 ms.

A right ventricular defibrillation lead with a DF-1 connector (Sprint Quattro) was positioned in the right ventricular apex and connected to the defibrillator generator. A right atrial pacing lead (CapsureFix Novus MRI) was placed in the right atrial appendage in a standard fashion. LBBAP was then performed using a lumenless pacing lead (SelectSecure 3830) delivered through a fixed-curve sheath (C315-His). During lead advancement, paced QRS morphology demonstrated late R in V1 with V6 R wave peak time of 81 ms, V6–V1 interpeak interval of 56 ms and QRS transition by threshold test, consistent with LBB capture. Finally, the Attain Performa was placed in posterolateral branch of coronary sinus. All leads were connected to Cobalt XT HF generator (the coronary sinus lead was connected to the left ventricular port and the LBBAP lead to the RV-P/S port after capping the IS-1 connector plug of the ICD lead) (Medtronic, Minneapolis, MN, USA) with biventricular paced QRS 124 ms (*Figure 2*). Therefore, in this setting, VT sensing and iATP delivery are performed through the LBBAP lead. Defibrillation threshold testing was successful at 20J. Final setting included VF zone at >188 beats/min and VT zone at >100 beats/min with iATP. Following optimal medical therapy, the patient was discharged home in NYHA functional Class I and was able to ambulate independently.

**Figure 3:**
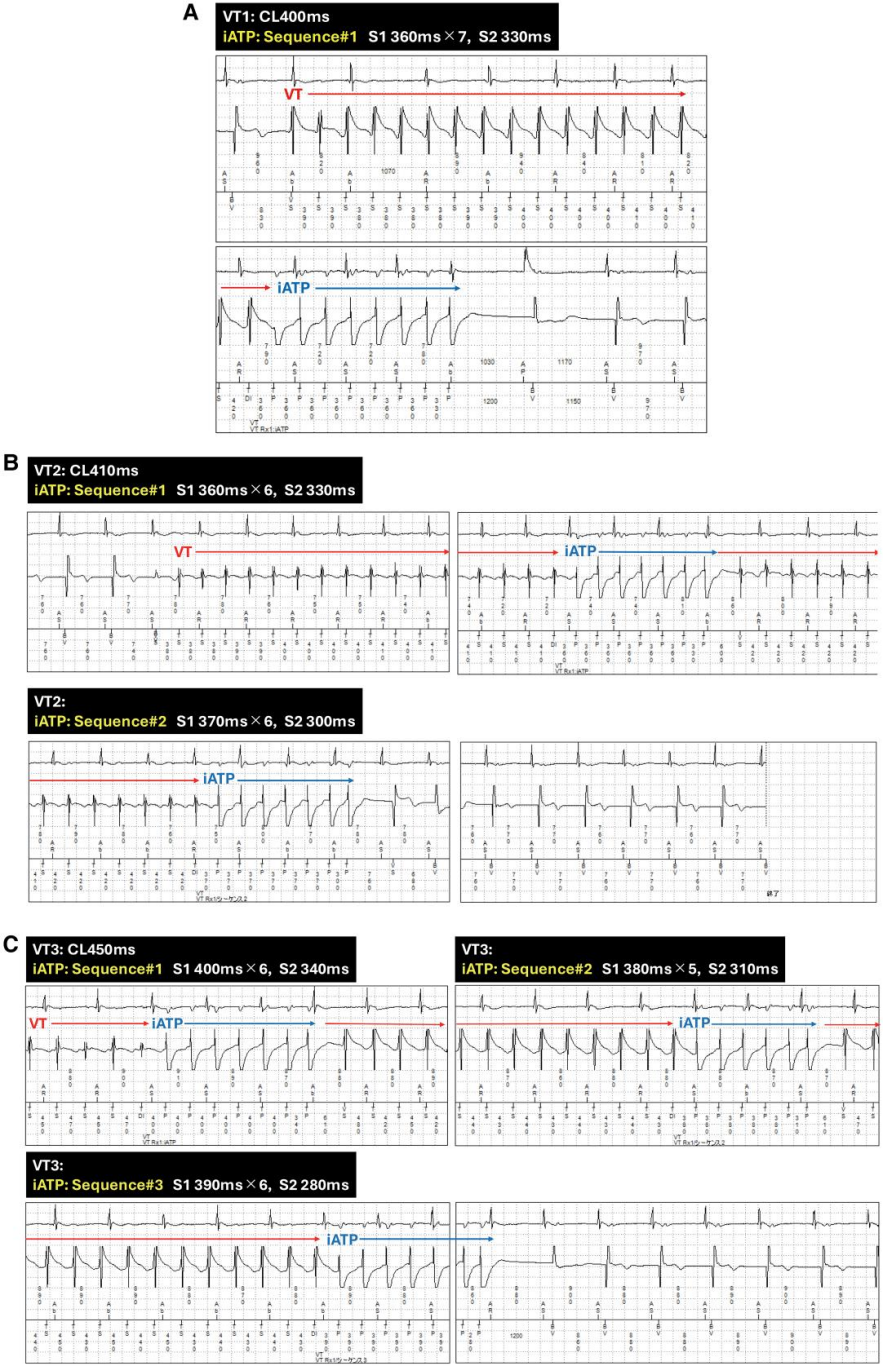
Intracardiac electrogram (EGM) of ventricular tachycardia (VT). (*A*) Intrinsic antitachycardia pacing (iATP) successfully terminates VT1 (CL: 400 ms) in the first sequence. (*B*) iATP failed to terminate VT2 (CL: 410 ms) in the first sequence. However, based on the post-pacing interval, the second sequence varied the series of the pulse of iATP and terminated VT2 in the second sequence. (*C*) iATP failed to terminate VT3 (CL: 450 ms) in the first and second sequences. The third sequence terminated.

One year after discharge, VT with a cycle length (CL) of 400 ms (VT1) occurred. The first sequence of iATP included 7 S1 pulses with a CL of 360 ms, followed by an S2 pulse with a coupling interval (CI) of 330 ms, leading to VT termination (*Figure 3A*). VT (VT2) occurred 1 month after VT1 episode, with a CL of 400 ms. The ventricular electrogram was different from that previously observed in VT1. The first sequence of iATP consisted of six S1 pulses with a CL of 360 ms, followed by an S2 pulse with a CI of 330 ms, which failed to terminate the VT. Based on the analysis of the post pacing interval of 600 ms, the second sequence of iATP was delivered with six S1 pulses, with a CL of 370 ms, followed by an S2 pulse with a CI of 300 ms, and VT was terminated (*Figure 3B*). VT (VT3) occurred 3 months after VT2, with a CL of 450 ms. The first sequence of iATP consisted of six S1 pulses with a CL of 400 ms, followed by an S2 pulse with a CI of 340 ms, which failed to terminate the VT. After first sequence of iATP, a change in VT morphology was observed, indicating a shift to a different re-entrant circuit. The second sequence of iATP was delivered with five S1 pulses, with a CL of 380 ms, followed by an S2 pulse with a CI of 310 ms, which failed to terminate the VT. The third sequence of iATP was delivered with 6 S1 pulses, with a CL of 390 ms, followed by an S2 pulse with a CI of 280 ms, which succeeded to terminate VT (*Figure 3C*). The patient did not undergo conventional ATP and shock therapy because all subsequent 16 VTs were appropriately terminated by iATP during the follow-ups that lasted 18 months (*Table 1*). The patient was continuously followed by remote monitoring. Following each VT episode, telephone interviews confirmed the absence of VT and iATP related symptoms. Successful VT termination by iATP prevented heart failure events and further clinical deterioration during follow-up.

**Table 1:** Summary of ventricular tachycardia events

VT event	CL (ms)	Detection	Therapy	iATP sequence	Acceleration	Termination
1	410	Appropriate	Appropriate	1	None	Yes
2	410	Appropriate	Appropriate	2	None	Yes
3	400	Appropriate	Appropriate	3	None	Yes
4	500	Appropriate	Appropriate	1	None	Yes
5	400	Appropriate	Appropriate	1	None	Yes
6	410	Appropriate	Appropriate	1	None	Yes
7	350	Appropriate	Appropriate	1	None	Yes
8	400	Appropriate	Appropriate	1	None	Yes
9	410	Appropriate	Appropriate	1	None	Yes
10	380	Appropriate	Appropriate	1	None	Yes
11	400	Appropriate	Appropriate	1	None	Yes
12	410	Appropriate	Appropriate	1	None	Yes
13	400	Appropriate	Appropriate	2	None	Yes
14	410	Appropriate	Appropriate	1	None	Yes
15	400	Appropriate	Appropriate	1	None	Yes
16	400	Appropriate	Appropriate	2	None	Yes

All 16 VTs were terminated by iATP therapy appropriately detected and delivered via LBBAP lead.

CL, cycle length; iATP, intrinsic antitachycardia pacing; LBBAP, left bundle branch area pacing; VT, ventricular tachycardia.

## Discussion

In this case, repeated VT episodes were consistently terminated by iATP delivered through the LBBAP lead following VT detection by the CRT-D system. Although ATP is traditionally delivered from an RV apical or septum lead,^4^ the present observation suggests that ATP delivery from an LBBAP lead may represent a feasible alternative approach for VT termination.

Conventional ATP delivered from the RV apex or RV septum terminates VT by introducing paced impulses into the re-entrant circuit during its excitable gap.^4^ The success of ATP therefore depends on whether pacing stimuli can reach and penetrate the tachycardia circuit. However, RV pacing sites are often anatomically distant from left ventricular re-entrant circuits. In such situations, pacing impulses may require longer conduction times to reach the circuit, which can reduce ATP efficacy or occasionally result in acceleration of VT. ATP delivered from septal sites has been shown to achieve higher VT termination rates compared with apical pacing. This advantage is likely related to shorter conduction distance and earlier penetration into the re-entrant circuit.^5^ In contrast, pacing from the LBB area may provide several theoretical advantages. LBB area captures or engages the His–Purkinje conduction system, allowing more rapid propagation of pacing stimuli throughout the ventricular myocardium in comparison to septal pacing.^2^ Recently, experimental evidence supports this concept.^6^ In a preclinical ischaemia-reperfusion VT model, ATP delivered to the LBB area achieved significantly higher VT termination rates than ATP delivered to the RV (70.2% vs. 47.3%, *P* = 0.040). In that study, electrophysiological mapping demonstrated that LBBA-ATP produced earlier activation of both Purkinje fibres and the working myocardium compared with RV-ATP, indicating that conduction system capture facilitates faster disruption of re-entrant circuits. These findings provide a plausible explanation for the successful VT termination observed in the present case. Importantly, the present case does not establish superiority of ATP delivered from an LBBAP lead over ATP from an RV apical or septum lead. Rather, it highlights a potential mechanistic advantage that warrants further investigation in larger cohorts.

Despite these potential advantages, several concerns remain. First, the long-term performance of conduction system pacing leads requires further investigation. Recently developed conduction system pacing lead platforms, including newer systems such as the OmniaSecure lead platform (Medtronic, Minneapolis, MN, USA), may have mechanical and electrical characteristics that differ from conventional pacing leads. The interaction between deep septal lead positioning and repeated ATP delivery has not yet been systematically studied.^7^ Second, optimal device programming when ATP is delivered from a conduction system pacing lead remains uncertain. Algorithms such as iATP dynamically determine pacing sequences based on tachycardia responses and may enhance VT termination; however, it remains unclear whether the optimal pacing parameters differ when pacing originates from the LBB area rather than the RV lead.^8^

In addition, the potential proarrhythmic effects of ATP delivered from conduction system pacing sites remain unknown. Because pacing impulses delivered near the His–Purkinje system can propagate rapidly through the conduction network, there is a theoretical risk that pacing stimuli may engage multiple exits of a re-entrant circuit or alter ventricular activation patterns in a manner that could accelerate tachycardia. Further studies are therefore needed to clarify the electrophysiological mechanisms, efficacy, and safety of ATP delivered from conduction system pacing leads.

This case suggests that iATP delivered from an LBBAP lead can effectively terminate VTs in a patient treated with LOT-CRTD. Conduction system pacing may provide a novel platform for ATP therapy, although further clinical studies are required to determine its optimal use and long-term safety.

Statement of consent: Informed consent was obtained from the patient in accordance with the Committee on Publication Ethics guidelines.

## Lead author biography


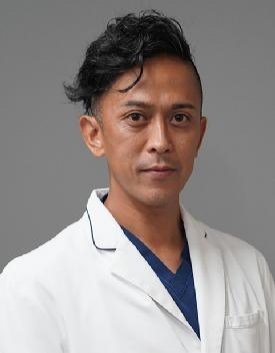


Noriaki Moriyama serves as the Director of Cardiac Implantable Electronic Device Therapy at Shonan Kamakura General Hospital, Japan.

## Data Availability

The datasets used and/or analysed during the current study are available from the corresponding author on reasonable request.
